# Coating of Silicone Monofilaments with Elastic Carbon Black-Silver-Silicone Layers and Their Characterization Especially with Regard to the Change of the Electrical Resistance in Dependence on Strain

**DOI:** 10.3390/polym14040806

**Published:** 2022-02-19

**Authors:** Kristina Klinkhammer, Ramona Nolden, Rike Brendgen, Manuela Niemeyer, Kerstin Zöll, Anne Schwarz-Pfeiffer

**Affiliations:** 1 Research Institute for Textile and Clothing (FTB), Niederrhein University of Applied Sciences, Webschulstr 31, 41065 Mönchengladbach, Germany; kristina.klinkhammer@hs-niederrhein.de (K.K.); ramona.nolden@outlook.de (R.N.); rike.brendgen@hs-niederrhein.de (R.B.); manuela.niemeyer@hs-niederrhein.de (M.N.); kerstin.zoell@hs-niederrhein.de (K.Z.); 2 Faculty of Textile and Clothing Technology, Niederrhein University of Applied Sciences, Webschulstr 31, 41065 Mönchengladbach, Germany

**Keywords:** carbon black silicone, conductive coating, metallic fillers, ultrastretchable sensor, resistive sensor

## Abstract

Smart textiles have properties that outperform the conventional protective and decorative function of textiles. By integrating special sensors into clothing, body functions and movements can be detected. Piezoresistive sensors measure a change in electrical resistance due to the application of force in the form of stretching, pressure or bending. In order to manufacture such sensors, conventional non-conductive textile materials need to be made conductive by finishing processes. Therefore, a non-conductive silicone monofilament was coated with a conductive carbon silicone and additional silver-containing components and investigated for its suitability as a strain sensor. The changes in electrical resistance and the gauge factor as a measure of the sensitivity of a sensor were measured and calculated. In this publication, the electrical properties of such a filament-based sensor in the context of particle composition and concentration are discussed. The electrical resistance was already significantly reduced in a first step by coating with conductive carbon silicone (145 kΩ). The addition of silver-containing components further reduced the electrical resistance in a second step. Thereby, flat flakes of silver proved to be much more effective than silver-containing particles (5 kΩ at 20% addition). The former was easier to integrate into the coating and formed contact surfaces with each other at higher concentrations. Stretching the samples increased the resistance by enlarging the distance between the conductive components. With 30% silver-coated glass flakes in the coating, the highest gauge factor of 0.33 was achieved. Consequently, the changes in electrical resistance during stretching can be exploited to detect motion and the gauge factor indicates that even small changes in strain can be detected, so the herein developed coated monofilaments are suggested for use as strain sensors. Future work includes matching the particle composition and concentration to the exact application and investigating the sensors in the field.

## 1. Introduction

The monitoring of various bodily functions and movements is an important field of application for smart textiles. The properties of these exceed the familiar functions of textiles, such as clothing, warming and protection, and combine these, for example, with intelligent functions using electronic components, processors, sensors or actuators [1]. Sensors have the ability to convert a specific stimulus into a signal that can be measured electronically [2]. Piezoresistive sensors detect the change in the electrical resistance of a component due to strain or deformation [3]. An important factor in assessing the sensitivity of strain sensors is the “gauge factor”, which describes the change in electrical resistance as a function of the change in length of the material [4,5]. Sensors that are used, for example, to monitor a patient’s movement patterns must be stretchable and flexible. Therefore, they must not restrict movement or interfere with comfort [5]. Due to their flexibility, sensors based on textiles are ideal for this purpose [6,7,8,9,10]. In order to measure the change in electrical resistance as a function of strain, a conductive material must be present. Therefore, classical piezoresistive strain sensors are made of metal foils, with a gauge factor of about 2–5 [11] and are thereby rather inflexible [12]. However, for motion measurement on different joints of the human body, such as knees or elbows, strain sensors that allow for and can detect a large strain are needed. Textiles based on polymers are suitable for this purpose. Since conventional polymers used for textiles are not sufficiently conductive, they must be modified by adding or integrating conductive material, which is referred to as extrinsic conductivity. This can be achieved, for example, by coating them with conductive coatings or integrating conductive fillers, such as carbon black [7,13,14,15], carbon nanotubes [16], or metallic additives [17,18,19] into the polymer matrix. Above a certain concentration of the added conductive fillers, the so-called percolation threshold, a transition from insulator to conductive material occurs and the conductivity of the material increases abruptly. The reason for this is the formation of conductive clusters or pathways in the polymer matrix that extend throughout the sample, starting when a certain conductive concentration is reached [20,21]. The required filler concentration here depends on the specific combination of conductive material and polymer and is usually 3–15 wt% [15,22]. The size and shape of the added fillers influence the filler density and thus also the conductivity [21,23,24].

Silver has the highest conductivity of all metals and is, therefore, ideally suited to improve the electrical properties of other materials. Synthetic fibres have already been functionalized with thin layers of silver [25,26], and the conductivity of *conductive adhesives* [27] or coatings of textiles has been increased by the addition of silver (particles) [24]. The conductivity of textiles can also be increased by combining different materials. In this way, the desired properties can be targeted and costs for expensive metals can be saved. In this publication, we present an approach to increase the conductivity of carbon silicone by adding silver components and we discuss the application of the particle-containing stretchable coating compound to an elastic filament by dip coating. In addition, the dependence of the electrical resistance on the form and concentration of the silver components is investigated.

## 2. Materials and Methods

### 2.1. Materials

Silicone monofilament (Muriel^®^ Grip) (LeMur S.r.l, Ala, Italy) with 2000 dTex (diameter 470 µm) was used for coating with the conductive silicone Elastosil LR3162 (components A and B; Wacker Chemie AG, Munich, Germany). The silicone oil Belsil DM 1 Plus (Wacker Chemie AG, Munich, Germany) was used to adjust the viscosity of the coating Webschulstrmixture. Various metal components were added to the coating mixture in different concentrations. Table 1 provides information on the added metal-containing materials.

### 2.2. Methods 

#### 2.2.1. Coating of Monofilaments 

The coating paste was prepared by manually mixing components A and B of Elastosil LR 3162 (Wacker Chemie AG, Munich, Germany) (ratio 1:1) with equal amounts of Belsil DM 1 Plus (Wacker Chemie AG). This coating of electrically conductive liquid silicone is defined as “carbon silicone” in the following text.

The conductivity was increased by the addition of metal particles in concentrations of 5–20%, or 5–40% (eConduct Glass silver 352000) to the finished coating paste before their application to the monofilaments. The given percentages refer to the total mass of the coating paste, not to the dry weight.

For coating the monofilaments, the coating paste was filled into the cylinder of a 5 mL syringe (B Braun, Melsungen, Germany) missing the syringe plunger. The syringe was vertically clamped in a tripod and the monofilament was manually pulled through the cone of the syringe with a dispensing needle (Ø 0.84 mm, Vieweg GmbH, Kranzberg, Germany). The coated monofilaments were cut at a length of 35 cm and subsequently fixed with tape into a needle bar. The coated monofilaments were free-hanging vulcanised in the oven for 5 min at 120 °C.

For each of the five monofilaments with the same treatment, the weight (analytical balance “ABS” from Kern, Lörrach, Germany) and length were determined with a calibrated ruler and, therefore, the linear weight was calculated in g/cm. The mean value was calculated from the results and the standard deviation was determined.

#### 2.2.2. Microscopy

For the optical and elementary analysis, light and laser microscopy but also scanning electron microscopy combined with energy dispersive X-ray spectroscopy were carried out.

(1) Light microscope

The uncoated and coated monofilaments were examined with the light microscope VHX-600 (computational module) and VH-Z250R (microscope) from Keyence (Osaka, Japan or Neu-Insenburg, Germany). To prevent strong light reflection due to the added metal particles, a polarizing filter was used below the objective lens to adjust the reflection intensity individually. The images were taken with the objectives of 20×, 30×, 50×, 100× and 150× magnification. 

(2) Laser microscope

The samples were also viewed with the VK-X100 laser microscope from Keyence (Neu-Insenburg, Germany). A special float system was used to stabilize the microscope during the measurements. The monofilament samples under investigation were rastered with the resolutions of 10×, 50×, and 100×. 

The laser directed at the sample scans the individually selected area of the sample and detects its surface, including unevenness and height differences. This is also summarised under the term surface roughness, which is a shape condition consisting of an uneven surface on which continuous peaks and valleys of different heights, depths and distances occur. 

The Multi-File Analyzer software from Keyence (Neu-Insenburg, Germany) was used to analyse the laser images. The software enables file management and image display. For each rasterized laser image, a 2D laser image, a coloured height image, and a 3D image are generated. The 3D image results from the laser optical 2D image and the analysed height image of the surface structure. 

(3) Electron microscope and EDX

The samples were examined using TM4000 Plus scanning electron microscope (SEM, Hitachi High-Technologies GmbH Europe, Krefeld, Germany). Images were taken at 30×, 100×, 150×, 300×, 1000× magnification. For the detection of elements on the sample surface, mapping was performed using energy dispersive X-ray spectroscopy (EDX) (Bruker SCU, Brucker Nano GmbH, Berlin, Germany). EDX mapping was performed at magnification 150× and samples were analysed for elements C, O, Si, Ag. For analysis, the distribution of atoms in atom [%] was evaluated and the mean and standard deviation of three measurements per sample were calculated.

#### 2.2.3. Electrical Resistance Measurements 

The coated and uncoated samples were subjected to an electrical-resistance measurement (Voltcraft LCR-300). Here, the samples were stretched from 150 mm to 200 mm and then released. The electrical resistance was measured in 10 mm steps. 

## 3. Results

### 3.1. Mass and Layer Thickness 

The uncoated monofilaments have an average linear weight of 0.0019 ± 0.0001 g/cm. The coating with carbon-silicone increases the linear weight of the monofilaments to 0.0030 ± 0.0002 g/cm and thus by approx. 57%. Due to the low total weight of the samples, the expected change in mass due to the addition of particles is within the range of variance of the measurement methods and no significant change in linear weight due to the addition of particles could be measured. 

### 3.2. Optical Evaluation of the Monofilaments 

For the qualitative assessment of the coating, the samples were examined microscopically. Light microscopy was used to obtain an overview of the sample. Laser microscopy and scanning electron microscopy were used to examine details. 

**Figure 1:**
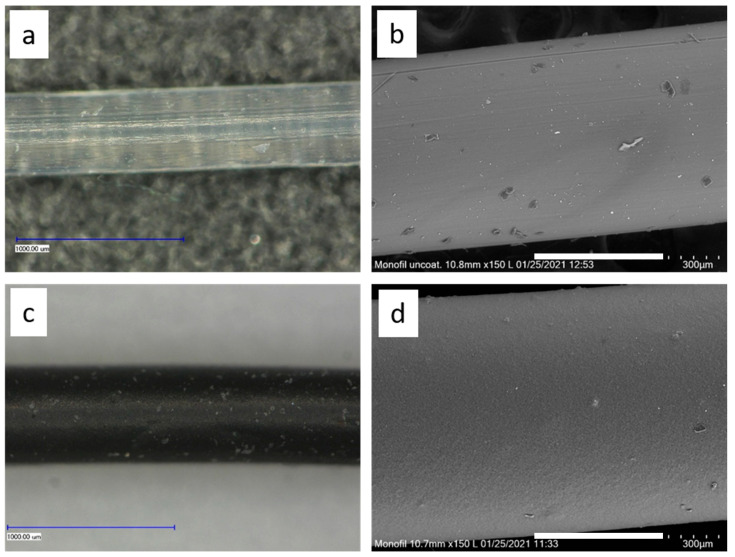
Photographs of the monofilament; left: Light microscopy; size bar 1 mm, right: SEM; size bar 300 µm; (**a**,**b**) monofilament uncoated; (**c**,**d**) monofilament with carbon-silicone coating.

The microscopy images in Figure 1 show the uncoated and the carbon-silicone coated monofilaments.

The uncoated monofilament is colourless and appears almost transparent under the light microscope. The SEM image shows slight soiling, probably due to dust, on the surface of the monofilament.

The coating colours the monofilament black. The light microscope shows white spots on the surface that cannot be identified more closely by the SEM. However, SEM imaging shows a uniform coating of the monofilament. A comparison of the diameters of the two samples shows an increase in diameter of approx. 100 µm.

### 3.3. Optical Evaluation of the Samples with Silver Components

**Figure 2:**
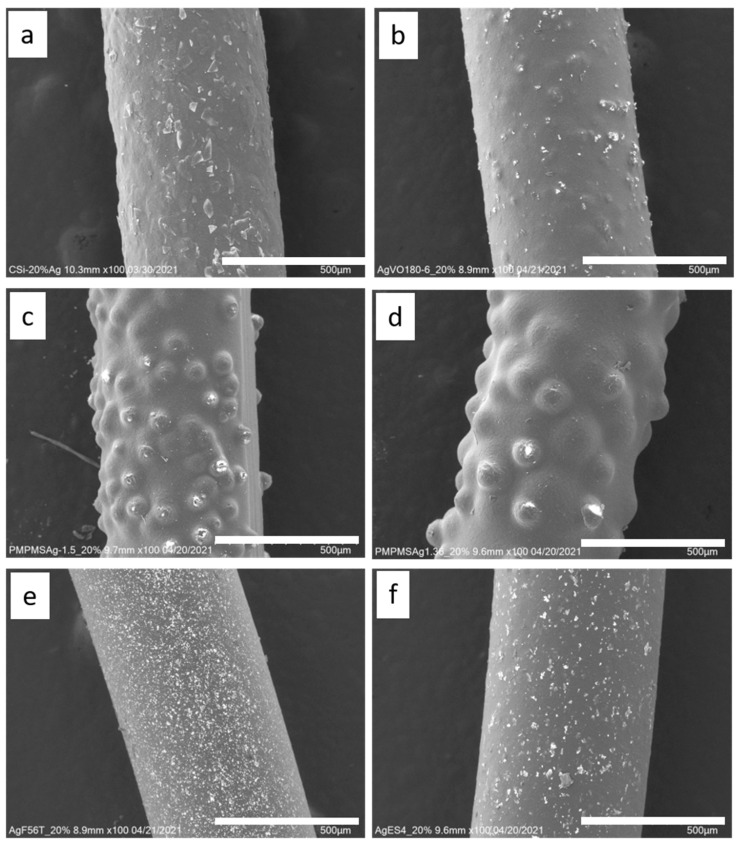
SEM images of coated monofilaments each with 20% silver addition. (**a**) Ag352000, (**b**) AgVO180-4, (**c**) Ag1.53, (**d**) Ag1.36, (**e**) Ag F56T, (**f**) AgES4. Size bar = 500 µm.

To increase the conductivity, various silver-containing additives were mixed into the coating paste (see Table 1). Figure 2 compares the coated monofilaments with silver particles/flakes.

The different shapes of the silver additions can be easily seen with the laser microscope. The Ag352000 flakes have a size of 10–65 µm and are distributed relatively evenly over the monofilament surface. In some cases, they protrude slightly from the coating. The AgVO180 particles are slightly smaller and better integrated into the coating due to their rounder shape. For the silver-coated PMMA spheres Ag1.53 (38–45 µm) and Ag1.36 (90–106 µm) the spherical shape is very visible and the size difference between the two materials becomes obvious. In some places, the carbon-silicone coating on the spheres appears to be destroyed. The flakes AgES4 (6–50 µm) and AgF56-T (3–16 µm) differ mainly in size. Both flakes are very evenly distributed over the surface and the roughness of the monofilament surface increases only slightly due to the integration of the silver flakes, which was observed visually.

**Figure 3:**
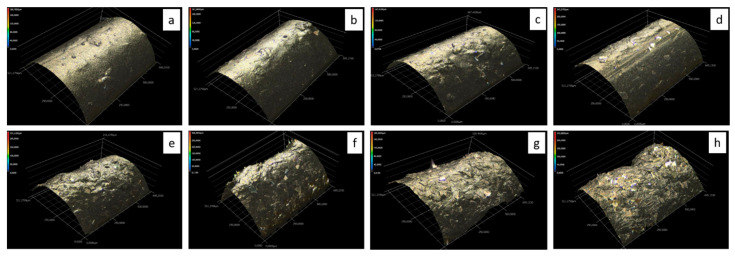
3D laser microscopy images of monofilament coated with carbon silicone and Ag352000, (**a**) 5%, (**b**) 10%, (**c**) 15%, (**d**) 20%, (**e**) 25%, (**f**) 30%, (**g**) 35%, (**h**) 40%.

In addition to the concentration, the shape and size of the added silver materials influence the surface roughness. This is particularly evident in the non-uniform shape of the Ag352000 silver flakes. Furthermore, Figure 3 demonstrates this using 3D laser microscope images.

**Figure 4:**
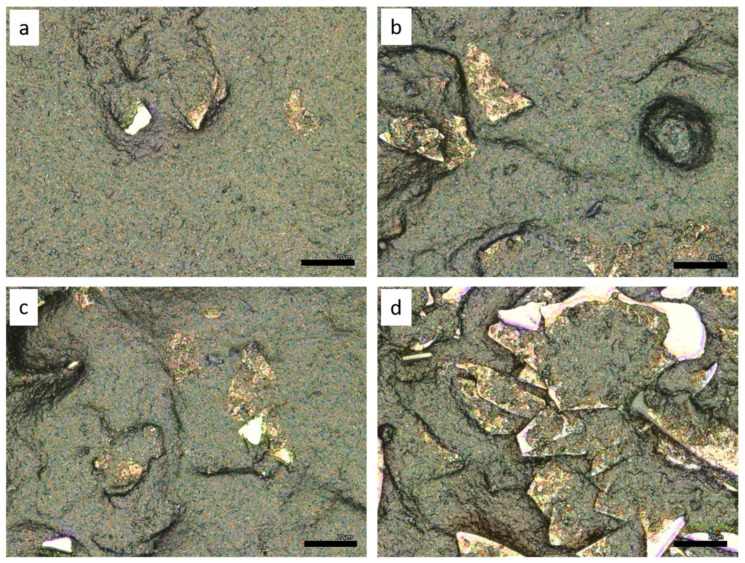
Detailed laser microscopy images of monofilament coated with carbon silicone and Ag352000, (**a**) 5%, (**b**) 25%, (**c**) 30%, (**d**) 40%; size bars 20 µm.

With an increasing concentration of silver flakes the density of the flakes on the monofilament surface increases, as does the roughness. The detailed photographs in Figure 4 illustrate this.

It is evident that at a silver flake concentration of 5% the flakes are scattered on the surface of the monofilament. At a concentration of approx. 25–30%, the density is so high that the flakes form a closed layer and contact each other. At 40% the flakes are clearly overlapping in several layers on the surface.

### 3.4. EDX Measurements

EDX mapping was performed on selected samples. This method provides information about the atomic composition of the sample surface. Table 2 shows the data of the EDX analysis of samples coated with carbon silicone and different silver-containing additives, each at a concentration of 20%.

The evaluation of the EDX measurements shows very different silver contents for the individual samples. For the flakes Ag352000, AgVO180 and the PMPMS particles approx. 0.16–0.2% silver was detected on the surface of the filaments. For AgES4, five times the amount (about 1%) and for AgF56T, 13 times the amount was measured with 2.62%. Table 3 shows the EDX analysis data for samples with increasing concentration of Ag352000 particles.

The evaluation of the EDX data shows that both the coating with carbon silicone and the integration of silver flakes Ag352000 have an effect on the atomic composition of the samples. The coating with carbon silicone increases the carbon content on the surface and reduces the silicone content. The integrated silver flakes can be detected optically via microscopy images (see Figure 3), as well as spectroscopically via EDX at the surface. The detected amount of silver on the surface increases with an increasing concentration of the coating paste. At 5 and 10% silver flakes in the coating, 0.1% silver was detected at the surface. At 40% silver flakes in the coating, about 0.9% silver was detected by EDX, i.e., about 10 times. The values given are mean values from three measurements for each concentration, with a high variation of standard deviations. The detected values of the silver content can only reflect a tendency, since the distribution of the flakes on the filament surface is not completely homogeneous (see also Figure 4). This affects the EDX values, especially at low flake concentrations. Furthermore, only the surface silver is detected. Flakes lying in the coating are not detected by the method. In addition, calcium was found in comparable quantities to silver. According to the manufacturer, these are components of the glass flakes that have been coated with silver.

**Figure 5:**
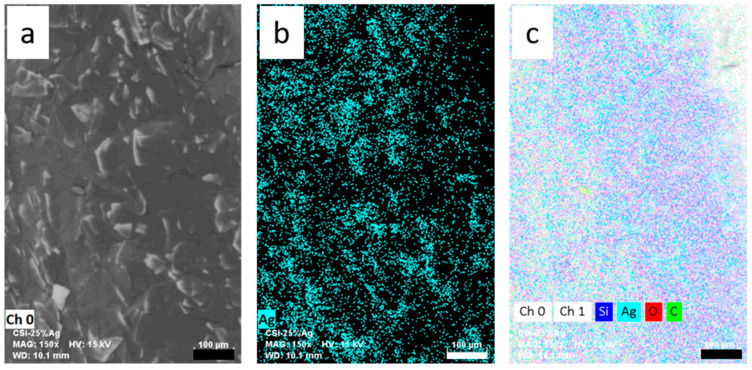
EDX mapping of Ag32000_25%; (**a**) SEM image; (**b**) Ag; (**c**) superimposed image of all relevant elements and SEM image. Size bar 100 µm.

The distribution of the silver flakes can be assessed via EDX mapping. Figure 5 shows a not-quite-uniform distribution of flakes in the coating.

### 3.5. Resistance Measurements 

**Figure 6:**
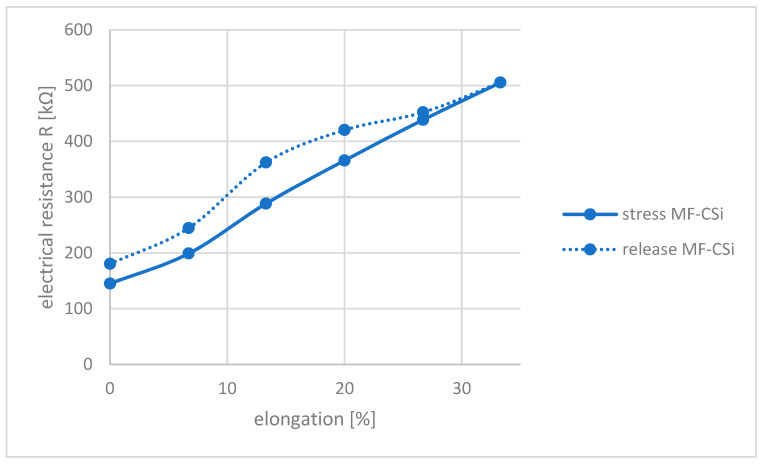
Electrical Resistance R as function of elongation for carbon silicone coated monofilament (MF-CSi) (length in relaxed state 150 mm).

Electrical resistance measurements were carried out to evaluate the conductivity of the monofilament. The uncoated monofilament showed infinite resistance. By coating it with carbon silicone, the resistance was reduced and various measurements were made. For this purpose, the monofilament was clamped at a length of 150 mm and then gradually stretched to 200 mm and electrical resistance was measured at every 10 mm stretch. Subsequently, the strain was released step by step and the resistance was measured again. A hysteresis was observed during the measurement under load (stretching) and without load. Figure 6 illustrates this using the example of monofilaments coated with carbon silicone. 

During stretching, the resistance increases continuously and a large increase from 150 kΩ to 505 kΩ can be observed. During unloading, the resistance decreases again. However, the same values as observed during strain are not obtained, but the values during unloading are always above the load values. This phenomenon was observed in all samples. When repeating the measurements the following day, the initial resistance had the same value as before. Thus, there is a recovery of the electrical resistance with time (data not shown). The largest hysteresis error is 10 mm in the length change and occurs at approximately 365 kΩ.

**Figure 7:**
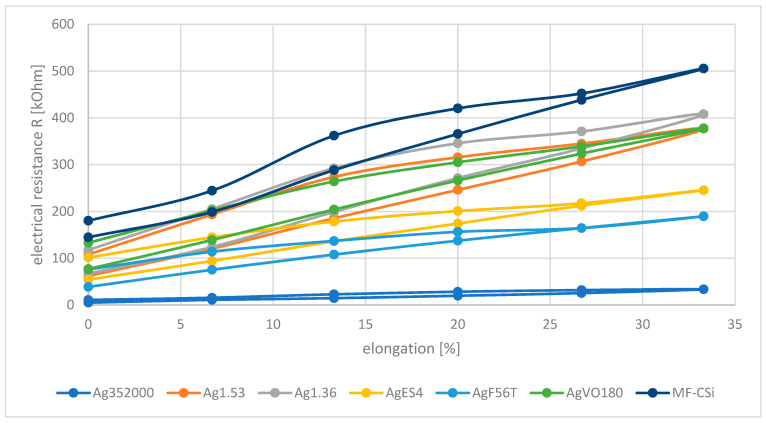
Electrical Resistance R as function of elongation for different silver additives (20%). The lower curves belong to stress, the upper curves belong to relaxation of the monofilament.

The different silver particles and flakes were added to the carbon-silicone coating to improve the conductivity. Figure 7 shows the influence of the particle type on the electrical resistance using the composition with a 20% addition. 

As observed with the original material, a hysteresis of the measured values also occurs here. The lower curves correspond to the strain and the upper curves to the unloading. The hysteresis errors are max. 10–14 mm for the various additives. Compared to the reference of the filament coated with carbon silicone (MF-CSi), the electrical resistance decreases for all samples due to the addition of silver. However, there are large differences in the values. The initial values without strain range from 5 kΩ (Ag352000) to 77 kΩ (AgV180). The maximum resistance is reached by the maximum strain. Here, the values increased to between 34 kΩ (Ag352000) and 408 kΩ (Ag1.36). This describes an increase in the electrical resistance of approx. 660–450%.

**Figure 8:**
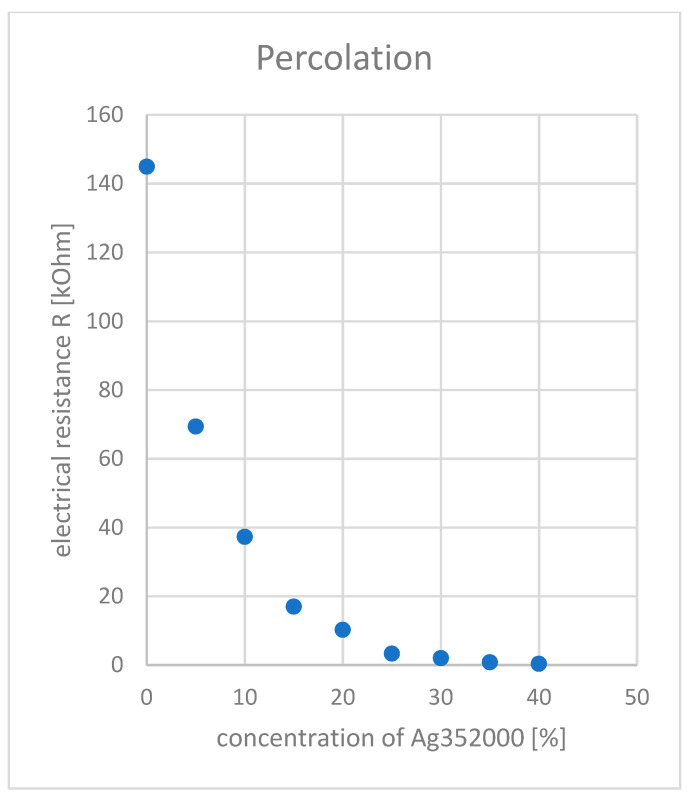
Electrical Resistance R as function of concentration of Ag352000 at 0% elongation.

For the desired application, the highest possible electrical conductivity and thus the lowest possible electrical resistance is desirable. Therefore, the product Ag352000, which showed the lowest resistance in the first investigations, was examined more closely. Concentrations of 5–40% of silver flakes were added to the carbon-silicone coating. Resistance measurements were also carried out with these samples, initially without strain (see Figure 8).

This showed an exponential dependence of the electrical resistance on the flake concentration. Up to a flake concentration of approx. 10%, the resistance drops significantly, from approx. 25% it gradually approaches a straight line and tends towards zero. The percolation threshold is thus in the range of approx. 5%, since the largest slope is observed here. 

**Figure 9:**
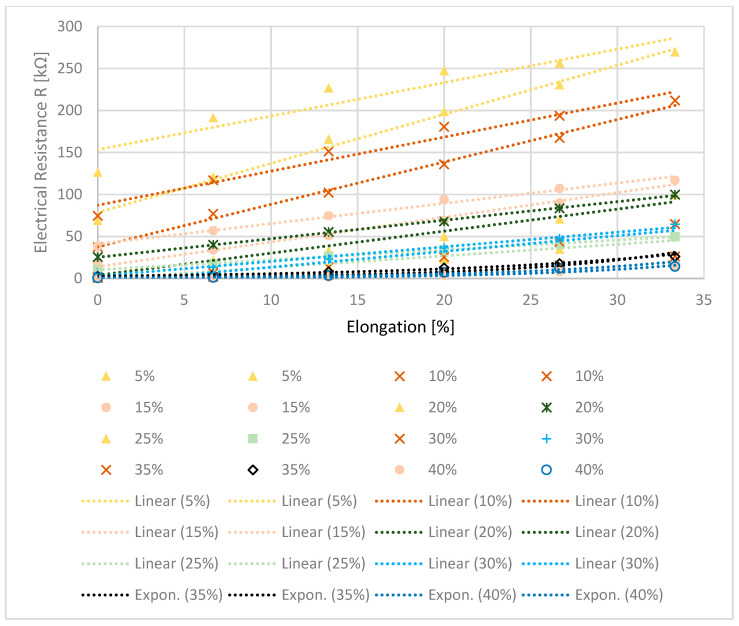
Electrical Resistance as function of elongation for different concentrations of Ag352000. The lower curve belongs to stress, the upper curve belongs to release of the monofilament.

Finally, the dependency of the electrical resistance on strain at different silver concentrations was also investigated. The results can be seen in Figure 9.

As already observed, a hysteresis of the measured values exists here as well. Here, again, the lower curves correspond to the strain and the upper curves to the relief. At low concentrations the resistance increases linearly with increasing strain, at higher concentrations from 35% the resistance increases exponentially. Here, an increase in the slope of the resistance due to strain is observed with an increasing flake concentration. At concentrations of 5 to 15%, the electrical resistance increases by a factor of 3 to 6, at 20 and 25% by a factor of 9 and 15, respectively, and at a concentration of 30% and above, even by a factor of more than 30.

## 4. Discussion

### 4.1. Visual Assessment and Silver Content

The microscopy images show very different properties for the mixed-in silver materials. Table 1 shows SEM images of the pure silver materials. It is noticeable that the manufacturer’s information on the morphology of the materials only allows for a rough classification. The differences only became clear by the microscopy images.

The Ag352000 material consists of silver-coated glass flakes that appear to be stiff and relatively thick. They also have straight edges. Due to these properties, they do not conform well to the curvature of the filament when integrated into the carbon-silicone coating, and the contours of the flakes are easily noticed on the surface of the monofilament (see Figure 2). Furthermore, there seems to be a preferred direction in the orientation of the flakes in the coating, whereby all of the microscopy images show predominantly the faces of the flakes rather than the edges. The detected silver content on the filament surface ranges from 0.1% (addition of 10%) to 0.88% (addition of 40%) for these flakes. If the flakes are evenly distributed in the coating, between 1/20 and 1/10 of the silver contained is therefore detected by EDX. From a concentration of 30% silver flakes in the coating, the amount of silver detected via EDX increases to 1/10 (see Table 3). The reason for this could be that from this concentration onwards the loading is so high that the flakes in the coating touch each other and form a coherent layer, as can also be seen in Figure 4.

In contrast, the AgES4 silver flakes appear thinner and more flexible. They have rather rounded edges, as can be seen in Table 1. As a result, they blend better into the coating and the surface appears more uniform (see Figure 2). The detected amount of silver on the surface of the filaments via EDX is about 1% (Table 2), which for the Ag352000 flakes, is 1/20 of the added amount. However, since the AgES4 flakes are 100% silver flakes, the absolute value is higher.

The diameter of the silver flakes of AgF56T is, at 3–16 µm, significantly smaller than the previously discussed flakes. These are also flakes consisting of 100% silver. The edges of the AgF56T flakes appear frayed and uneven. Due to the small diameter, these flakes distribute very well in the coating and the surface of the monofilaments is homogeneous. A silver content of 2.6% (see Table 2) was detected in these monofilaments via EDX. This corresponds to a content of 1/10 at a flake concentration of 20% in the coating. Due to their smaller size, and thus their lighter weight per flake, a larger number of flakes are added in absolute terms at the same weight concentration than for the larger flakes. Since the surface area of smaller objects is larger in comparison to their volume, the higher detected silver content can be explained by this circumstance.

According to the manufacturer, the other materials tested are microspheres or particles. Both Ag1.36 and Ag1.53 are microspheres made of poly(methyl methacrylate) (PMMA) coated with silver. The silver content of both is <18% and they differ in size and density. Visually, the different sized microparticles do not differ. Both exhibit an almost perfect spherical shape. The surface is slightly textured and resembles the structure of a golf ball (see Table 1). The microscopy images of the coated filaments in Figure 2 show that the surface is strongly structured by the addition of the microspheres. This is also due to the fact that at least the larger Ag1.36 spheres have a diameter of 90–160 µm, which is larger than the layer thickness of the carbon-silicone coating. This would require the spheres to be in a monolayer around the filament. However, due to the relatively low concentration of 20% by weight, a complete layer of spheres around the filament is not achieved. The situation is similar for the smaller Ag1.53 microspheres. Although their diameter of 38–45 µm should be smaller than the carbon silicone layer thickness, there seems to be a non-continuous monolayer of sphere on the filament surface here as well. The silver content detected by EDX is very similar for both spheres, 0.16 and 0.2%, respectively (see Table 2), and corresponds to about 1/20 of the added amount of silver.

According to the manufacturer, AgVO180 consists of particles made of 100% silver. However, the microscopy image in Table 1 shows that these are relatively non-uniform spherical structures with a wide size distribution of 36–63 µm. The particles were uniformly embedded in the carbon silicone coating and well covered by the coating. This also explains the low detected silver content of 0.2% (see Table 2). This corresponds to only 1/100 of the added amount of silver. 

Overall, a similar silver content was measured on the surface of the filaments for all particles. For the flakes, the amount of silver detected was dependent on the size and type of flake.

Theoretically, a higher loading of the coating mass of the monofilament with silver components would be possible [23]. However, the lowest possible loading should be preferred, as higher loading amounts result in various negative effects, such as an increase in production costs, deterioration of mechanical properties (e.g., embrittlement, high viscosity) or problems during processing (e.g., due to clogging of nozzles) [21].

### 4.2. Electrical Conductivity

#### 4.2.1. Monofilament with Carbon Silicone Coating

Textile components are suitable for use as sensors for detecting human movement patterns due to their stretchability and physiological properties [7,8]. However, for use as piezoresistive sensors that detect strain due to the subject’s movement, the textile components must be conductive. Conventional textiles and the polymers they are made of are insulators and do not conduct electric current [8]. This is also true for the silicone filament that serves as the basis for the present experiments. By coating it with conductive carbon silicone, the conductivity is significantly increased and the electrical resistance decreases from infinity (polymer = insulator) to 145 kΩ (see Figure 6). Piezoresistive sensors are suitable for the detection of movement due to their property of representing strain by changing resistances. When strain is applied, the spacing of the fillers required for the conductivity of extrinsic polymers increases and the resistance increases. At a certain spacing, the percolation threshold is exceeded and the resistance increases exponentially. For accurate measurements, the largest possible change in electrical resistance is required even at small strains. As can be seen from Figure 6, the electrical resistance increases by approximately 350% from 150 kΩ to 500 kΩ at a strain of 50 mm. When the sample is unloaded again, a slightly lower resistance is measured at a 150 mm clamping length than at the start of the measurement and hysteresis is observed. This is typical for a resistive textile sensor [7] and occurs in systems where the data output depends on both the load and the history of the load. Reasons for this can be friction and rearrangements in the material due to the initial load [28]. Hysteresis can cause errors in the evaluation of the data, since a resistance value can be assigned to different strains. This must be considered when evaluating the data. The error due to the hysteresis amounts to a maximum of approx. 14 mm.

#### 4.2.2. Integration of Silver into the Coating

For processing the sensor data, it is advantageous to have the lowest possible electrical resistance [8]. This can be achieved, for example, by integrating metal-containing particles into the coating of the monofilament [23]. With 20% by weight of silver components in the coating, a significant reduction in electrical resistance was sometimes achieved, as shown in Figure 7. At this concentration, the processing properties were not negatively affected.

The resistance curves in Figure 7 illustrate clear differences in the ability of the silver components used to increase the conductivity of the coating. The three silver particles investigated (AgVO180, Ag1.53 and Ag1.36) behave almost identically. They exhibit similar high resistance values, both without load and with load. This can already be estimated from the microscopy images of the samples, which show no contact of the particles within the coating (see Figure 2). The conductivity achieved by adding electrically conductive fillers depends not only on the concentration of the filler, but also on its shape and distribution [23]. Especially in the case of spherical fillers, possible contact areas are very small and the microparticles tested proved to be rather unsuitable for producing good conductivity at moderate filler concentrations due to their shape [21,24]. 

Elongated or flat fillers can be better integrated into a coating and can form larger contact areas with each other [16,24]. By this, lower filler concentrations are sufficient to achieve high conductivities, or lower resistivities are achieved with the same concentrations as for particles. This phenomenon was observed for the integrated silver flakes. They all show lower resistances than the particles. Among them, the silver coated glass flakes Ag352000 show the lowest electrical resistances. In contrast to the pure silver flakes AgF56T and AgES4, they arrange themselves very parallel to the monofilament surface due to the inflexible glass base and thus offer the best conditions for overlapping/contact of the flakes with each other and good conductivity (see Figure 2).

The greater the change in resistance compared to the change in length, the more accurate the sensing. The addition of the silver components results in an increase in the resistance change, as shown in Table 4. The gauge factors for an elongation of the monofilament of 33% (corresponding to elongation from 150 mm to 200 mm) are also given here.

Again, the advantages of the Ag352000 flakes are evident. They have the largest percentage change in resistance and the largest gauge factor at 33% elongation. These properties led to a closer examination.

#### 4.2.3. Different Concentrations of 352000

The percolation threshold for extrinsically conductive polymers describes the sudden increase in conductivity at a certain concentration of conductive fillers in the polymer [21]. In the case of the integrated silver flakes Ag352000, the percolation threshold is located at approx. 5% addition (see Figure 8). Starting at the percolation threshold, there are enough conductive elements embedded in the coating to allow for current flow to occur. Increasing the flake concentration above the percolation threshold leads to better conductivity and a reduction in electrical resistance. There is an exponential dependence in the investigated range between 5 and 40% addition. The microscopy images in Figure 4 show an actual overlapping of the silver flakes from a concentration of approx. 25% onwards. Here, the electrical resistance is only 3 kOhm (referred to a length of 150 mm). Increasing the contact area, as it occurs at higher filling levels, further reduces the resistance. It is expected that above a certain filler concentration, saturation occurs and a further increase in concentration does not lead to a further increase in conductivity. This condition was not reached in the performed experiments. However, it would also not make sense in the present system, since with 40% silver addition, considerable difficulties already occurred in the homogeneous mixing and in the application of the coating paste and the monofilaments became increasingly brittle.

**Figure 10:**
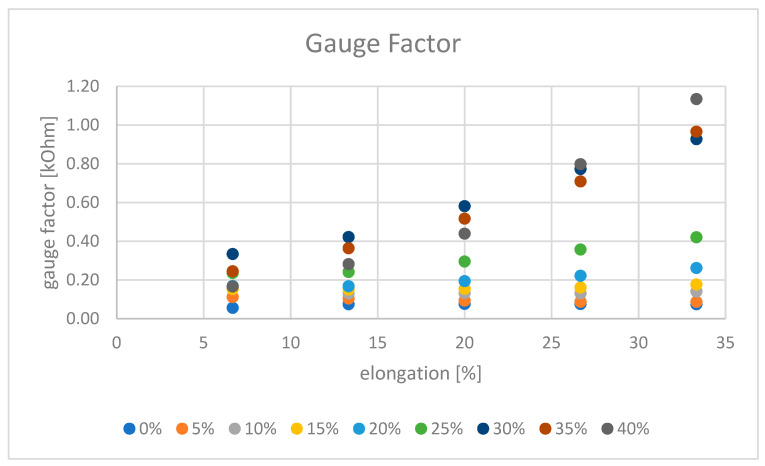
Gauge factors for different concentrations of Ag35200 flakes in dependency on elongation.

For the use of the coated monofilament as a sensor, the change in electrical resistance with the strain is an important criterion. Stretching increases the distances between the conductive silver components, thus reducing the number of electrical connections and increasing the resistance [29]. This effect is shown in Figure 9, where at lower flake concentrations, a linear relationship between strain and resistance change can be observed, at 35 and 40% this relationship becomes exponential. Strain at high flake concentrations results in similar electrical states as at low flake concentrations without strain. A change in the percolar system takes place [8]. Depending on the concentration of the conductive filler material and the extent of the strain, it is possible that the strain will undercut the percolation threshold and the material will no longer be conductive. This must be taken into account for possible application in the field. While the absolute change in electrical resistance is higher for lower concentrations, the percentage increase in resistance increases for higher concentrations (see Table 5). As described earlier, the gauge factor is a measure of the sensitivity of the sensor and it describes the change in resistance with strain. Table 5 illustrates the relationship for the experiments performed, while Figure 10 visualizes this.

At low concentrations, the gauge factor remains almost constant over the strain (Figure 10). Therefore, a linear relationship between electrical resistance and strain can be observed (see also Figure 9). At higher flake concentrations, the absolute value of the gauge factor is higher and it also increases with increasing strain—so there exists not a linear but an exponential relationship between resistance and strain (Figure 10), as can also be seen in Figure 9. The larger the gauge factor, the higher the sensitivity of the sensor, as small changes in strain cause a larger change in resistance, making it easier to detect [30]. For the presented system, this means that the highest possible concentrations of silver flakes should be chosen to build an accurate sensor. However, both the higher material costs and the expected negative influence on the mechanical properties [21] have to be considered when adjusting the silver concentration. A good solution here is a silver flake concentration of 30%. Here, an overlapping of the flakes in the coating takes place and the highest gauge factor without strain is achieved. In addition, a linear relationship between resistance and strain and a small hysteresis lead to a good evaluability of the measurement data. Measurement of the viscosity of the coating pastes with different flake concentrations and a correlation with reasonable application methods for a higher throughput might be useful. In this way, the step towards an automated application process could be taken. In addition, it would make sense to investigate different coating thicknesses in the future, which would allow for further optimization of the conductivity.

## 5. Conclusions

Monofilaments made of silicone are not suitable for the production of piezoresistive sensors due to their insulator properties. The electrical resistance of the used monofilaments could be significantly reduced by coating with a conductive carbon-containing silicone. However, only the addition of conductive components with or consisting of silver reduced the electrical resistance to such an extent that metrological processing of the data is meaningful. Due to their spherical shape, silver-containing microparticles showed clear disadvantages here. They were more difficult to integrate into the coating and also had very small contact areas, which led to a reduced current flow. Flat flakes could be better integrated into the coating and, in particular, less flexible glass flakes with a silver coating aligned themselves parallel to the monofilament axis. This resulted in larger contact areas between the flakes and a significant reduction in electrical resistance. For a sensor with high sensitivity, it is necessary that even small changes in strain can be detected. A measure for this is the gauge factor (GF). This was shown to be dependent on the type of components added and their concentration. A coating with 30% glass flakes proved to be particularly effective. Here, a GF of 0.33 was achieved and a low resistance was measured both with and without strain loading.

## Figures and Tables

**Table 1:** Information on added filler materials.

Product Name	Conduct Glass Silver 352000	Silver AGP V0180-4	PMPMS-AG 1.53	PMPMS-AG 1.36	Silver F56T	Silver ES-4
Code	Ag352000	AgVO180	Ag1.53	Ag1.36	AgF56T	AgES4
Producer *	Eckert GmbH	Doduco GmbH	Cosperic LLC	Cospheric LLC	Doduco GmbH	Doduco GmbH
Product description	flakes	particles	microbeads of PMMA with silver coating	microbeads of PMMA with silver coating	flakes	flakes
Size D10-D90 [µm] **	10.0–65.0	36–63	38–45	90–106	<3–16	6–50
Silver content [%] **	app. 18	≥99.9	<20	<20	≥99	≥99.7
REM images (500× magnification)	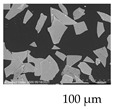	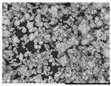	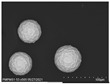	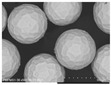	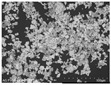	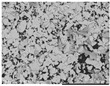

* Eckert GmbH, Hartenstein, Germany; Doduco GmbH, Pforzheim, Germany; Cospheric LLC, Santa Barbara, USA; ** according to manufacturer’s data.

**Table 2:** EDX analysis of samples with silver containing coating (20% addition).

Sample	C [At.%]	O [At.%]	Si [At.%]	Ag [At.%]
Ag352000_20%	58.6 ± 0.4	27.6 ± 0.2	13.4 ± 0.3	0.2 ± 0.0
AgVO180_20%	58.3 ± 1.4	27.2 ± 0.5	14.3 ± 1.0	0.2 ± 0.1
Ag1.53_20%	60.2 ± 1.2	26.1 ± 0.9	13.4 ± 0.4	0.2 ± 0.1
Ag1.36_20%	55.9 ± 2.3	29.5 ± 1.8	14.4 ± 0.7	0.2 ± 0.1
AgF56T_20%	53.8 ± 5.9	28.1 ± 2.5	15.5 ± 2.9	2.6 ± 0.5
AgES4_20%	57.2 ± 1.9	27.5 ± 1.0	14.2 ± 0.6	1.0 ± 0.4

**Table 3:** Evaluation EDX analysis of samples with Ag352000 in different concentrations. The values represent mean values over three measurements. In some cases further elements, e.g., nitrogen, were detected. In this case, no new normalization was performed, but the output values were used.

Sample	C [At.%]	O [At.%]	Si [At.%]	Ag [At.%]	Ca [At.%]
uncoated monofil	48.4 ± 3.3	34.7 ± 1.3	16.9 ± 2.2	0	0
monofilament with carbon silicone	59.8 ± 0.6	26.6 ± 0.4	13.3 ± 0.5	0	0
Ag352000_5%	59.4 ± 0.0	26.4 ± 0.2	14.1 ± 0.2	0.1 ± 0.0	0
Ag352000_10%	57.9 ± 0.9	27.3 ± 1.2	14.8 ± 1.3	0.1 ± 0.0	0
Ag352000_15%	59.8 ± 0.7	26.1 ± 1.1	13.9 ± 1.4	0.2 ± 0.0	0.3 ± 0.1
Ag352000_20%	58.6 ± 0.4	27.6 ± 0.2	13.4 ± 0.3	0.2 ± 0.0	0.2 ± 0.0
Ag352000_25%	57.4 ± 1.6	27.3 ± 0.8	14.5 ± 0.8	0.4 ± 0.1	0.4 ± 0.1
Ag352000_30%	57.4 ± 1.8	27.6 ± 0.6	14.1 ± 1.5	0.5 ± 0.1	0.5 ± 0.0
Ag352000_35%	58.9 ± 0.9	27.3 ± 0.2	12.8 ± 0.6	0.5 ± 0.1	0.5 ± 0.1
Ag352000_40%	57.0 ± 2.3	28.0 ± 0.9	13.2 ± 1.0	0.9 ± 0.2	1.0 ± 0.3

**Table 4:** Increase in electrical resistance due to 33% elongation of monofilament as a function of added silver component.

Silver Component (Addition of 20%)	Increase in Electrical Resistance with Elongation by 33% [%]	Gauge Factor at 33% Elongation [1/%]
without	349	0.07
Ag352000	674	0.17
AgVO180	491	0.12
Ag1.53	607	0.15
Ag1.36	609	0.15
AgF56T	489	0.12
AgES4	452	0.11

**Table 5:** Gauge factor as a function of strain and concentration of Ag352000 flakes.

Flake Concentration [%]/Clamping Length [mm]	150	160	170	180	190	200	Increase in Electrical Resistance at Elongation from 0% to 33% [%]
0%	-	0.06	0,.7	0.08	0.08	0.07	349
5%	-	0.11	0.10	0.09	0.09	0.09	389
10%	-	0.16	0.13	0.13	0.13	0.14	568
15%	-	0.15	0.15	0.15	0.16	0.18	688
20%	-	0.17	0.17	0.19	0.22	0.26	971
25%	-	0.24	0.24	0.30	0.36	0.42	1501
30%	-	0.33	0.42	0.58	0.77	0.93	3189
35%	-	0.24	0.36	0.52	0.71	0.96	3316
40%	-	0.17	0.28	0.44	0.80	1.13	3878
**Elongation [%]**	0.0	6.7	13.3	20.0	26.7	33.3	

## Data Availability

The datasets generated during and/or analysed during the current study are available from the corresponding author on reasonable request.
